# HIV Status and Associated Clinical Characteristics Among Adult Patients With Cancer at the Uganda Cancer Institute

**DOI:** 10.1200/JGO.17.00112

**Published:** 2017-11-16

**Authors:** Rachel Bender Ignacio, Matine Ghadrshenas, Daniel Low, Jackson Orem, Corey Casper, Warren Phipps

**Affiliations:** **Rachel Bender Ignacio**, **Corey Casper**, and **Warren Phipps**, Fred Hutchinson Cancer Research Center; **Rachel Bender Ignacio**, **Matine Ghadrshenas**, **Daniel Low**, **Corey Casper**, and **Warren Phipps**, University of Washington; **Corey Casper**, Infectious Diseases Research Institute, Seattle, WA; and **Jackson Orem**, Uganda Cancer Institute, Kampala, Uganda.

## Abstract

**Purpose:**

HIV increases cancer incidence and mortality. In Uganda, the HIV epidemic has led to an elevated incidence of AIDS-defining cancers (ADCs) and non–AIDS-defining cancers (NADCs). Limited information exists about how frequently HIV infection complicates the presentation and manifestations of cancer in sub-Saharan Africa.

**Methods:**

We abstracted medical records from patients with cancer who were age 18 years or older who registered at the Uganda Cancer Institute from June through September 2015 to determine the burden of HIV. We used χ^2^ tests and generalized linear models to evaluate factors associated with HIV positivity. A sensitivity analysis estimated HIV prevalence in those untested.

**Results:**

Among 1,137 patients with cancer, 23% were HIV infected, 48% were HIV negative, and 29% had no recorded HIV status. Of those with recorded HIV status, 32% were HIV positive. Forty-two percent (149 of 361 patients) with ADCs were documented as HIV infected (51% of those with documented status) compared with 14% (108 of 776 patients) of those with NADCs (21% of those with documented status). In multivariable analysis, HIV infection was associated with ADC diagnosis (adjusted prevalence ratio [aPR] compared with NADC, 2.2; 95% CI, 1.5 to 3.0), younger age (aPR, 0.9 per decade increase; 95% CI, 0.8 to 1.0), and worse performance status scores (aPR, 1.2 per point ECOG increase; 95% CI, 1.0 to 1.5). When sensitivity analysis accounted for undocumented HIV status, the expected prevalence of HIV infection was 29% (range, 23% to 32%), and almost one fourth of expected HIV cases were undiagnosed or unrecorded.

**Conclusion:**

The prevalence of HIV infection among Ugandan patients with cancer is substantially higher than in the general population. Patients with cancer and HIV tend to be younger and have poorer performance status. Greater awareness of the dual burden of cancer and HIV in Uganda and universal testing of patients with cancer may improve outcomes of HIV-associated malignancies.

## INTRODUCTION

People infected with HIV have an increased risk of developing cancer.^1,2^ Most data about HIV and cancer comorbidity have been generated from studies of high-income countries, although the primary burden of the epidemic falls on low- to middle-income countries.^1,3^ The problem of comorbid HIV and cancer in sub-Saharan Africa (SSA) is greater than in high-income countries because of the higher prevalence of HIV, the limited resources to diagnose and treat patients, and the higher prevalence of oncogenic infections.^3,4^ Three cancers are identified as AIDS-defining cancers (ADCs): Kaposi sarcoma (KS), non-Hodgkin lymphoma (NHL), and cervical cancer. The incidence of many non-ADCs (NADCs; ie, cancers not considered ADCs) also is increased with HIV.

Knowledge of HIV status is essential to manage the treatment of patients with HIV-associated malignancies (HIVAMs). Early antiretroviral therapy (ART) has been shown to reduce cancer mortality in HIV-infected patients with KS and NHL.^1,5,6^ However, in countries with high ART coverage, the incidence of some NADCs continues to increase.^6,7^ Although ART coverage in SSA has improved during the past decade,^5^ the incidence of ADCs clearly is not decreasing in Uganda.^8^ The Joint United Nation Program on HIV/AIDS (UNAIDS) estimate for HIV prevalence in the Ugandan general population (limited to people age 15 to 49 years old) is 7.4%, and it varies by region from 4% to10%.^9^ The risk of mortality has been 2.3 times higher for selected HIV-infected patients with cancer^1^; except for ADCs, though, there is little consensus on how to treat comorbid cancer and HIV.^10^ This gap results from a lack of basic epidemiologic data on HIVAMs in high-prevalence regions, such as SSA. To address this gap, we sought to determine the burden of HIV infection and its association with disease presentation among patients with cancer at the Uganda Cancer Institute (UCI), which is the country’s sole national cancer center and which serves a catchment area of approximately 100 million people in Uganda and adjacent regions of South Sudan, Kenya, Tanzania, Rwanda, and the Democratic Republic of Congo.

## METHODS

We conducted a cross-sectional medical records review at the UCI. We used the UCI registration log to identify medical records of all patients age 18 years and older who registered for care at the UCI across 4 months in 2015. We excluded records from patients with benign diagnoses or with cancer recurrence rather than primary presentation. We abstracted demographics, laboratory and clinician written reports of HIV status, clinical data about HIV and cancer, and basic laboratory data about the intake visit. Data were captured in REDCap (Institute for Translational Health Sciences, Seattle, WA) and were analyzed in STATA V13.0/14.0 (Statacorp, College Station, TX).

When clinical and histopathologic diagnoses differed, we relied on histopathologic diagnoses. We used National Comprehensive Cancer Guidelines, or other society guidelines, to assign appropriate TNM and I through IV staging for each tumor type; AIDS Clinical Trials Group staging was used for KS.^11-29^ Because complete staging with imaging or surgery often is not available in this setting, we also classified tumors as early, late, or unstageable on the basis of available data (ie, documentation of distant lymph nodes or metastases qualified as late stage). When staging was possible, we classified stages I to II as early cancer and stages III to IV as late cancer. Hematologic cancers and KS were similarly classified as early or late according to disease-specific criteria. KS was defined as early stage if T0S0 or T1S0 criteria were met; otherwise, it was defined as latestage.^27,30^ Functional status was evaluated by Eastern Cooperative Oncology Group (ECOG) scoring from 0 to 5, in which a score greater than 2 was defined as poor functional status.^31^

We calculated binomial proportions and used χ^2^ tests to evaluate differences in HIV testing and status. We used generalized linear models with binomial or Poisson assumptions and robust standard errors to generate prevalence ratios. Because HIV results were presumed to be missing not at random, we only included persons with documented HIV status in prediction models. In multivariable models, we included univariable predictors with a *P* value ≤ .10 and those variables selected a priori (age, sex). When there was collinearity between predictors, we chose the best-fitting model that used a single one of these collinear variables, as determined by Akaike information criteria.^32^

To estimate the prevalence of HIV among patients who did not have HIV status recorded in the medical record, we assumed that missing data of HIV results was both nonrandom and dependent on the test result itself. Because certain patients might have been more likely to be tested for HIV, and thus have positive HIV results recorded, this bias might have overestimated the observed HIV prevalence. We performed a sensitivity analysis by imputing missed HIV diagnoses in weighted age, sex, and cancer type strata in those who did and did not have a recorded HIV status. When no HIV occurrences were recorded in a small stratum, we used the general Ugandan HIV prevalence (7.4%), weighted by regional representation of patients with cancer (7.9%), as the stratum prevalence. We then estimated unobserved (unrecorded/missed) HIV diagnoses for each stratum and compiled these with observed diagnoses.

This study was approved by the Fred Hutchinson Cancer Research Center Institutional Review Board, the National AIDS Research Committee, and Uganda National Council on Science and Technology. Informed consent was waived.

## RESULTS

Between June 1 and September 30, 2015, 1,456 new patients were registered at the UCI. We analyzed records for 1,137 after the following patients were excluded: those younger than 18 years (n = 161) or those with benign hematologic diagnoses (n = 17), recurrent cancers (n = 2), or blank charts (n = 22); 117 additional medical records were not locatable after several attempts. Patients had been in care for a median of 54 days (interquartile range [IQR], 47 to 61 days) at the time of chart abstraction. The median age was 49 years (IQR 37 to 72 years), and 56% of patients were women (Table 1). Forty-five percent of patients came from Central Uganda (including 23% from the capital, Kampala), 53% came from other regions in Uganda, and 2% came from neighboring countries. Less than 2% of charts lacked a clinical tumor diagnosis, and 88 charts (8%) lacked histopathologic confirmation of tumor type. Percent agreement between clinical and histopathologic diagnosis was 90.6% (κ = 0.90). The most frequent diagnoses were cervical cancer (n = 202; 17%), breast cancer (n = 127; 11%), and KS (n = 114; 10%). Sixty-four percent of charts contained sufficient data for staging, among which 76% of tumors were late stage (III to IV or equivalent; Table 1). Overall, 47% of solid tumors could be assigned to stage I through IV on the basis of information available in the chart.

**Table 1:**
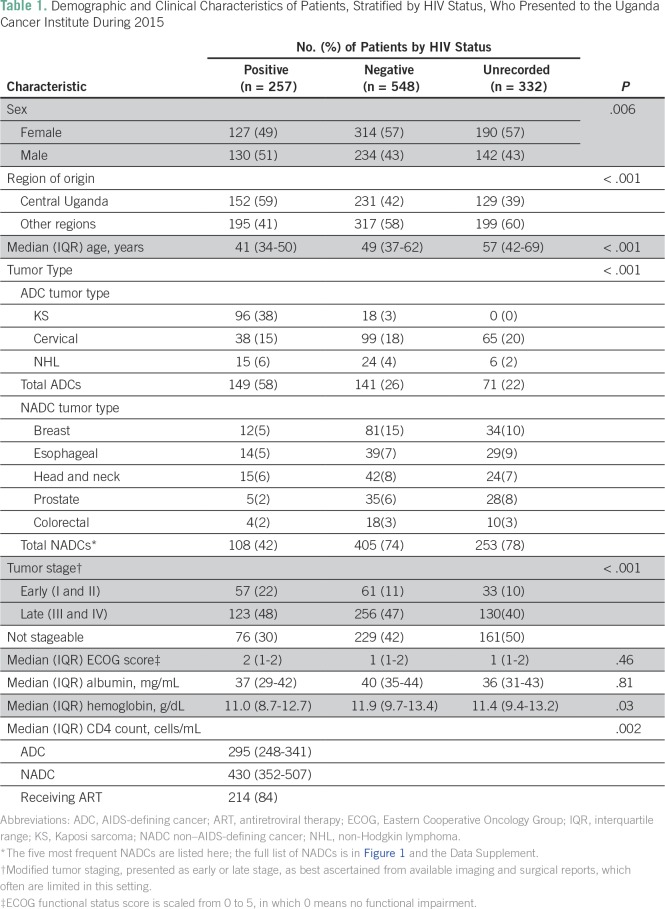
Demographic and Clinical Characteristics of Patients, Stratified by HIV Status, Who Presented to the Uganda Cancer Institute During 2015

### HIV Status

Of the 1,137 patients included in the study, HIV status was positive in 257 (23%), negative in 548 (48%), and unrecorded in 332 (29%) of patient records (Table 1). Among the 805 patients with a recorded HIV status, 257 (32%) were HIV positive. Approximately half of HIV occurrences had a documented date of HIV diagnosis; 7% were newly diagnosed within 3 months of UCI registration, and 42% were diagnosed more than 3 months earlier. CD4 T-cell counts were documented in 59% of those with reported HIV infection, and median CD4 count were 311 cells/mL^3^ (IQR, 124 to 503 cells/mL^3^). The median CD4 count for those with an ADC was 295 cells/mL^3^ (IQR, 248 to 341 cells/mL^3^) and for those with an NADC was 430 cells/mL^3^ (IQR, 352 to 507 cells/mL^3^; *P* = .002). Eighty-three percent of HIV-infected people were reportedly receiving ART,14% were not receiving ART, and 3% lacked documentation about ART.

Men were more likely to have HIV (prevalence ratio [PR], 1.2; 95% CI, 1.0 to 1.5; *P* = .04; Table 2): their HIV prevalence was 36% compared with 29% for women. HIV prevalence peaked in the fourth decade of life at 44%; no HIV infections were detected in people younger than 20 years or age 80 years or older. When age was restricted to 18 to 49 years for comparison with the Uganda UNAIDS population prevalence, the HIV prevalence was 40% (95% CI, 35% to 44%). For each increasing decade of life, the HIV PR was 0.8 (95% CI, 0.8 to 0.9; *P* < .001; Table 3). HIV prevalence was higher in patients from Central Uganda (30%) than in patients from other regions (25%; PR, 1.6; 95% CI, 1.3 to 2.0; *P* < .001). People with HIV were less likely than those with a documented HIV negative status to have late-stage cancer (75% *v* 87%; PR, 0.9; 95% CI, 0.8 to 0.9; *P* < .001). HIV was more common among people who had a poor functional status (35% in ECOG > 2 *v* 26% in ECOG ≤ 2; PR, 1.5; 95% CI, 1.0 to 2.0; *P* = .08). The prevalence of HIV was lower for each increasing quartile of hemoglobin on intake laboratories (PR, 0.8; 95% CI, 0.7 to 0.9; *P* < .001). Similarly, for each quartile increase of serum albumin, the PR of HIV was 0.8 (95% CI, 0.7 to 0.9; *P* = .001). Fewer people who were HIV positive lacked a histopathologic diagnosis for cancer (5%) compared with those who were HIV negative (7%) and those whose HIV status was undocumented (11%; *P* = .02). Similarly, people with HIV were less likely to have discordant clinical and pathologic tumor diagnoses (5%) than those who were HIV negative (10%) and who had an unrecorded HIV status (12%; *P* = .03). KS was no less often confirmed by pathology than other cancer types (4% v 8%; *P* = .14).

**Table 2:**
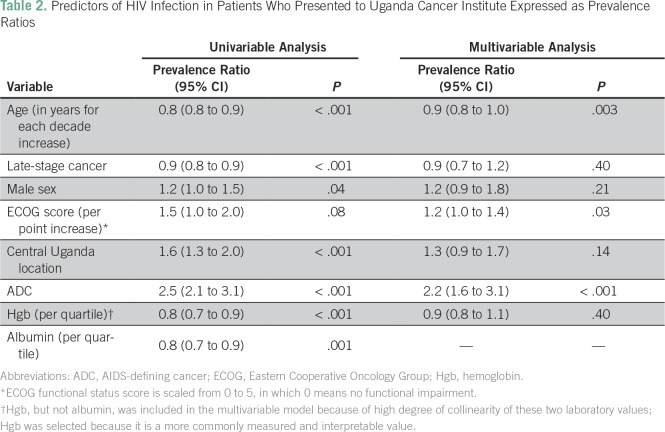
Predictors of HIV Infection in Patients Who Presented to Uganda Cancer Institute Expressed as Prevalence Ratios

**Table 3:**
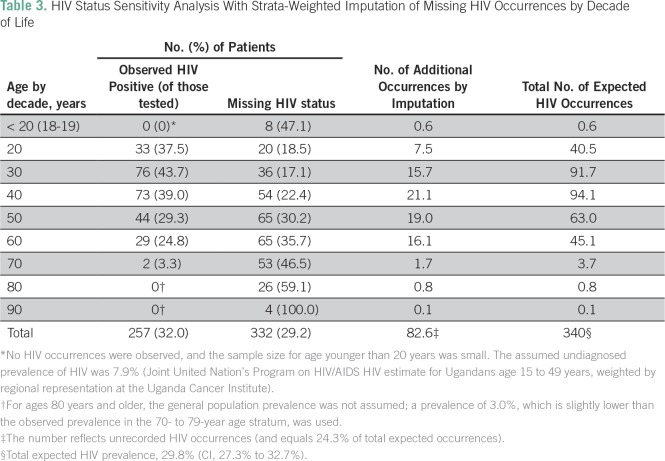
HIV Status Sensitivity Analysis With Strata-Weighted Imputation of Missing HIV Occurrences by Decade of Life

**Fig 1:**
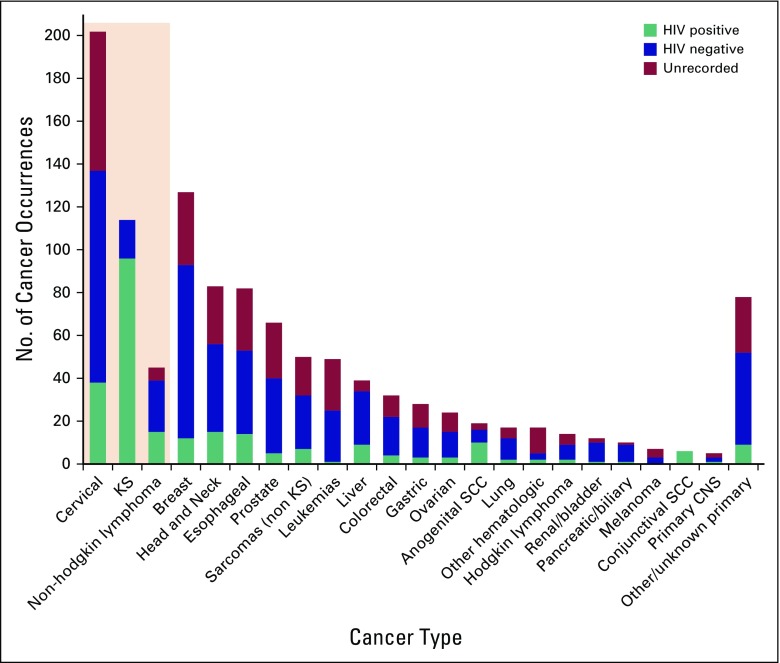
HIV status, including unrecorded HIV status, across individual tumor types in patients who presented to the Uganda Cancer Institute during 2015. Shaded box includes the three AIDS-defining cancers: cervical cancer, Kaposi sarcoma (KS), and non-Hodgkin lymphoma. Anogenital squamous cell cancer (SCC) includes vulvar, vaginal, penile, and anal cancers; cervical SCC is listed separately.

When the 114 occurrences of KS—within which the HIV prevalence was 84%—were exluded, the reported HIV prevalence in all other cancer types was 24% (95% CI, 21% to 27%). Among people with ADCs, 42% had documented HIV infection (84% of KS, 39% of NHL, and 28% of cervical cancer occurrences), 21% were HIV negative, and the remainder had no HIV status reported. Among people with NADCs, 14% had documented HIV infection, 52% were HIV negative, and 33% had no documented status; HIV prevalence among all people who had NADCs and a documented status was 21% (95% CI, 17% to 24%). HIV prevalence in people with HIV documentation who had some common NADCs included the following: 26% in liver cancer, 26% in esophageal cancer, 26% in head and neck cancer, 18% in colon cancer, 17% in lung cancer, and 13% in breast cancer (Fig 1). People who had an ADC had a PR of 2.5 for HIV compared with those who had an NADC (95% CI, 2.1 to 3.1; *P* < .001).

Age, sex, ECOG, geographic region, cancer category (ADC *v* NADC), modified cancer stage (early *v* late), and hemoglobin were included in the final multivariable analysis of factors associated with HIV infection (all *P* < .1 or selected priori). Among those with documented HIV status, only functional status, ADC, and age remained associated with HIV infection (Table 2).

### Estimates of Overall HIV Prevalence That Account for Bias in Missing Data

HIV status was unrecorded in 332 (29%) of 1,137 charts. In univariable analysis, each decade of age increase was associated with a lower probability that HIV status was recorded (*P* < .001). Men and women were equally likely to have unrecorded HIV status (30% each; *P* = .42). Only 20% of people with ADCs had an unrecorded HIV status, whereas 33% with an NADC lacked HIV documentation (*P* < .001). Within each cancer type, HIV documentation varied considerably: 100% of patients with KS had an HIV status recorded, but 32% of cervical cancer patients did not, despite the ADC diagnosis. HIV documentation ranged more widely among NADC types (Fig 1; Data Supplement).

Three sensitivity analyses were used to better define HIV prevalence in this population. First, weighted imputation was used for sex bias in HIV status documentation to estimate that 29% of expected HIV diagnoses were unrecorded, for an overall HIV prevalence of 32% (95% CI, 29% to 35%) in the entire cohort. Imputation that was based on decade of life estimated that 24% of expected HIV occurrences were unrecorded, for an overall HIV prevalence of 30% (95% CI, 27% to 33%; Table 3). A third estimate imputed HIV prevalence for each tumor type (observed range, 0% to 100%). This last method estimated that 21% of expected occurrences were unrecorded, for an overall prevalence of 29% (95% CI, 26% to 32%; Fig 1; Data Supplement). Two additional extreme conditions were considered: complete bias in HIV status recording, such that only HIV-negative people had an unreported status; and, alternatively, no bias in HIV documentation. With no unrecorded HIV diagnoses, the HIV prevalence would have been 23%. With no bias in documentation of HIV, the overall prevalence would equal the observed prevalence at 32%. Because tumor diagnosis is heavily associated with both age and sex, in which some cancers exclusively or predominately occur in one sex or age group, the most accurate HIV prevalence estimate was considered to be the one stratified by cancer diagnosis, although this method afforded less precision. Therefore, the estimate of the overall HIV prevalence in the UCI cancer population was 29% (range of estimates, 23% to 32%).

## DISCUSSION

To our knowledge, this is the first study in SSA to comprehensively evaluate the prevalence of HIV testing and HIV infection among patients who initiate cancer treatment. This study also examined the predictors of HIV infection among Ugandan patients with cancer as well as the association of HIV infection with morbidity.

We found that the HIV prevalence of all-comers to a national cancer institute in SSA was high. Even the most conservative sensitivity analysis estimated the HIV prevalence among patients with cancer at the UCI to be three-fold higher than the national HIV prevalence; more realistic assumptions produced a prevalence more than four-fold this estimate.^9^ Notably, UNAIDS country estimates pertain to the general population, age 15 to 49 years. Because the median age of patients who present to the UCI is 49 years, fully one half of patients with cancer are older than the population included in the UNAIDS estimate. There are no published age-stratified estimates for HIV prevalence in older Ugandan adults, but the HIV prevalence in UCI patients younger than 50 years old (40%) was five-fold the UNAIDS statistic applicable to that age group. Because the observed HIV prevalence in our cohort decreased by decade after age 40 years, we expect that HIV would also remain overrepresented in older patients with cancer compared with an age-matched general population. In addition to the high prevalence of HIV, as expected among patients with ADCs, a high prevalence of HIV among patients with NADCs, including in cancers more often associated with aging, was detected.

A limited number of biologic and demographic factors was associated with HIV infection among Ugandan patients with cancer. Patients with HIVAMs were younger and presented with earlier-stage cancers, but they had lower functional statuses and biologic markers of health (hemoglobin, albumin) compared with non–HIV-infected patients. In the multivariable analysis, HIV infection remained associated with younger age, decreased functional status, and an ADC diagnosis. Because patients with KS were more likely than those with any other tumor to present with early-stage disease, and because most patients with KS were HIV positive, this correlation in part may explain why HIV-infected people were more likely to present with early disease. Poorer functional status and biomarkers could reflect nutrition and comorbid infections. Data from the United States and Europe demonstrate that people with HIV infection are diagnosed at a younger age than HIV-negative comparators, but this observation in part may be due to differences in age distributions of HIV-positive and HIV-negative populations.^33,34^ Our observation that the median age of HIV-positive patients is younger than that of HIV-negative patients in part may be due to a younger HIV-positive population overall, although Uganda lacks population data to support a standardized risk analysis to evaluate whether this is an observational bias.^35^ Earlier-stage presentation and a higher rate of tumor histopathology confirmation may have reflected expedited cancer detection and referral for people engaged in HIV care in a country where primary/preventive care is uncommon except for those who attend the HIV clinic. The high ART usage rate and the median CD4 count also support the hypothesis that referrals to cancer treatment favor patients with previously diagnosed HIV who are already engaged in care, or possibly those patients who caregivers felt were more likely to benefit from cancer therapy. Adults with NADCs were much less likely than those with ADCs to have HIV results recorded; a full one third of patients with NADCs lacked an HIV status in their charts, despite an HIV prevalence of 21% in those with results. When HIV is not diagnosed, or when it is known but not recorded, patients with HIVAMs may not receive additional considerations for coordinated HIV and cancer care. For example, in addition to the WHO recommendation that all HIV-infected people receive ART regardless of CD4 count, patients with cancer may need expedited ART referral to facilitate immunologic support during chemotherapy. In addition, on the basis of CD4 count or drug-drug interactions, oncologists might choose alternate anticancer agents or modify dosing or timing with ART initiation, regardless of whether the cancer is considered associated with HIV.

This study has some notable limitations. First, the design of the study was a retrospective medical records review. There may have been recording bias in the data, which could lead to a higher proportion of patients with unrecorded HIV results than truly unknown status if people who were truly HIV negative were less likely to have their status recorded in their medical record. We also lacked complete data about cancer staging and other relevant clinical details in many charts, which hindered the ability to draw conclusions about clinical factors associated with HIVAM presentation. People with blank or unlocated charts (< 10% total) may have had different characteristics than those with completed charts, including the possibility of early death after registration. Finally, analyses categorized tumors as NADC or ADC because of the small numbers of individual cancer types; consequently, we were unable to draw conclusions about individual cancer types. The strengths of this study include a large sample size with near complete ascertainment of all adult patients who received cancer care at the only referral center to serve this region of East Africa; this minimized availability bias in a population with large geographic spread and high mortality.

To our knowledge, this is the first study to evaluate HIV prevalence and clinical characteristics of HIVAM among all patients at a cancer treatment center in SSA. The findings highlight the strong relationship of these two epidemics in SSA. Before cytotoxic chemotherapy is provided, it is important to know the HIV status of a patient so that ART can be initiated as appropriate and so that ART and chemotherapy regimens can be modified and monitored as necessary. In addition, diagnosis of HIV in patients with cancer can improve care coordination and minimize adverse events related to immunosuppression, drug-drug interactions, and opportunistic infections. Up to one third of all adult patients with cancer are estimated to be HIV positive, which suggests that universal HIV screening during intake to a cancer center may be beneficial. Although the HIV prevalence and incidence of individual cancers in the general population vary across SSA, these results emphasize the importance of universal HIV testing and treatment in all clinical settings, particularly in patients with cancer who are from HIV-endemic settings.
